# Queer occultism, sentimental biopower, and becoming ‘bottoms’ as a means to divest from white supremacy among practitioners of magic in Montréal

**DOI:** 10.1177/13675494231225659

**Published:** 2024-02-04

**Authors:** Sydney Sheedy

**Affiliations:** Concordia University, Canada

**Keywords:** Affect, astrology, bottoming, decolonization, divestment, impressibility, occult, queer, sentimental biopower, whiteness

## Abstract

Astrology, magic, and other psychic healing practices are undergoing a cultural revival, notably among those on the Left who employ it as a language for social justice. Queer practitioners have claimed kinship with the occult through a perceived shared abjection, deeming it an inherently queer resource for self- and community empowerment, and naming anti-racism and decolonization key aims of their work. At the same time, these forms of occultism draw suspicion, not least among practitioners themselves, who are critical of the ways these knowledge traditions have been complicit in ‘spiritual genocide’. Drawing from ethnographic fieldwork with 30 informants in Montréal in 2022, I investigate the occult’s appeal among queer people as a process of affective expansion, wherein practitioners attune to heretofore repressed lifeways, knowledges and worlds that machineries of empire have rendered invisible. If contemporary occult movements represent a turning towards putatively repressed modalities that may rival those which we have otherwise inherited, queer informants claim a special relationship to these objects through a framework of sensitivity that magic allows them to workshop. Theorizing the occult as a biopolitical affect regime, I argue that informants ironically invest in historically racialized language of impressibility as indexes of social health at the same time that they locate queerness, rather than whiteness, as a conduit for that affective expansion. I argue that white informants demonstrate a particular anxiety about how to learn to become open to this otherwise, positing ‘bottoming’ as a spiritual and political imperative to become receptive to forms of accountability, reparations and solidarity. How does the occult represent an attempt to build capacity for receptivity among participants, and how do they link this capacity to the healing of white supremacy and decolonization?

In the past few years, there has been a major resurgence of magic, witchcraft and mystical healing practices. Whether on sale as ‘witch starter kits’ (Miller, 2022), in the development of apps for astrological compatibility, in published guides for modern witches, or in political movements to hex white supremacists (Stardust, 2021), practitioners call on ancestral wisdom in the form of magical resistance. More than a profitable market, many have noted its social progressivist bent, wherein occult practices like astrology have gained currency among people on the Left (Burton, 2019), and queer people more specifically (Beusman, 2015). Some writers have gone so far as to name the occult as *inherently* queer, wherein abject forms of knowledge and ritual become available to those whose experience of social dislocation predisposes them to those formulations (Dockray, 2018; Lee, 2011). According to Winstanley-Smith (2021), ‘This form of modern astrology [. . .] has become a resource of language and thought-shapes, even a kind of auxiliary practice for political activism, especially among queer-identifying persons and their allies’ (p. 104). In my own fieldwork, participants consistently refer to the occult as a location for social critique which is familiar to queer people by virtue of their marginality. According to one white, trans informant I interviewed,

I do think witchcraft is kind of inherently queer. I think it’s inherently nonconformist. Not everyone who does witchcraft or magic is queer, but they’ve always kind of been on the social margins. [. . .] So they’re always kind of removed from society and are respected, sometimes feared. And that’s a very queer experience as well, being different from the group somehow and being excluded, but also having this knowledge that other people may not have. (Interview, April 2022)

In the spring and summer of 2022, I undertook ethnographic fieldwork in Montréal, conducting three group and 12 individual interviews with 30 participants who all responded to the prompt: ‘What does it mean to claim rejected knowledge practices, like astrology and tarot, as forms of queer politics? What would it take to disrupt its historical whiteness?’ (Recruitment poster, 2022). Inclusion criteria for the study comprised identifying as queer, above 18, living in Montreal, and being a practitioner of ‘tarot, astrology, or other forms of psychic healing’. More than 80 people responded, and of the 30 that I interviewed, 20 identified as white, and 26 as settlers. Thirteen were transgender, and ages ranged from 23 to 46. I follow the lead of informants and use different terms to describe the umbrella of practices that they brought to the table. Some used the term ‘magic’, others were more likely to talk about witchcraft or spirituality. I tend to use the term ‘occult’ because of its connotation as a knowledge system that rivals the epistemological monopoly of science as a modern pursuit (Hanegraaff, 2014; Josephson-Storm, 2017), representing a knowledge relation more than a given set of tenets or beliefs, which helps me to articulate its engagements with power. On the recruitment website and in interviews I was transparent about my own position as someone who also ‘dabbled’ in astrology and tarot, who was raised with an interest in Pagan and feminist spirituality, but that my curiosity in this case is more epistemological. Participants were paid a small honorarium to participate, and five co-facilitators were hired and trained in ethics and data privacy to help lead group discussions, provide peer support if needed, and weigh in on research design.

Key to my broader project is an investigation of the ways that the occult has become available to claim as *always already* counterhegemonic, or the putative rupture of a system the querent wishes to challenge, whether this is articulated as white supremacy, capitalism or heteronormativity. Recognizing the ways that precursors to the contemporary phenomenon, such as New Age spirituality, have been charged as ‘spiritual genocide’ (Crowley, 2011), wherein white people especially find the means to resolve social ills through cultivating indigenous roots (Morgensen, 2011), I have been motivated to explore the racial stakes of this phenomenon and how participants themselves reflect on their locations within that assemblage. I see this phenomenon as part of a much broader negotiation of inheritance, historical reparations and the question of divestment from violence that looms large in the cultural imagination, especially for those living in settler colonies like Canada. In navigating the dissolution of previously enduring forms of identity and lineage, to what extent does the seeking out of some putatively ancient wisdom tradition represent a necessary betrayal of white supremacy and empire? Can these historical legacies really be circumvented or interrupted, as so many practitioners allege, and what risks are incurred in claiming inheritance of something else?

Since beginning this project in 2018, there has been a little academic writing on the correlation between psychic healing and queer political imperatives. Winstanley-Smith (2021) takes a political theology approach to evaluate the political potential of astrology, claiming that ‘The queer astrological model of self-understanding and self-analysis has potential [. . .] But is it political? And if it is, how so?’ While I think these are valid questions to ask, my approach takes seriously practitioners as social theorists in their own right, highlighting their own critical reflections on the efficacy of their practices. By undertaking ethnography, I engage with how practitioners themselves navigate the political stakes of their encounters: its ‘range of affects, ambitions, and risks’ (Sedgwick, 2002: 150). In their PhD thesis, Harris Kornstein (2021: 150) uses a digital media theory standpoint to argue that ‘queer enchantment’ operates as a form of queer technology that runs counter to other data-driven consumer media, promoting ‘mystical or intuitive forms of being in and making sense of the world’. Like Kornstein, I am interested in the ways in which practitioners claim the occult as a form of queer strategizing, however, my approach is more focused on the racial terrain out of which this relationship emerges. Kornstein names whiteness as central to the cultural formulation of witchcraft, but this observation serves as the end of their analysis rather than the beginning. On the ‘current trend in witch infatuation’, Lou Cornum (2018) writes, ‘I am sympathetic to this appeal even as I am suspicious of it; it marks a desire to be contrary to the colonial project, even if it does not always enact it’. These critical suspicions are rarely followed by a sustained attempt to explore how practitioners are actually negotiating that risk themselves, nor a consideration of what exactly makes witchcraft appealing as an apparently anti-colonial location.

In this article, I draw from fieldwork in order to explore *impressibility* as a key aspect of the queer occult, which I argue lies at the heart of its conceptions of social transformation and health. I use impressibility following Kyla Schuller’s (2018: 3) theorization, which refers to a body’s capacity to ‘be alive to movements made on it’, which is foundational to its political claims to life. Informants often apprehend magic as a process of building the capacity to affect or be affected by the world, described in terms of ‘sensitivity’ or ‘attunement’, which they see as crucial to divesting from taken for granted systems of power. I argue that exploring magic in terms of this rubric of impressibility brings into relief the racial stakes of the phenomenon, beyond oft-limiting debates about cultural appropriation. As such, race emerges as a theory of inheritance wherein white supremacy’s requisite forgetting, or incorporation of Otherness, makes fraught terrain for seeking out forms of social organization that might have existed ‘before’ it. The occult, as I hope to show here, operates as a negotiation of this terrain, including to what extent the cultivation of sensitivity might offer a way to heal from the afterlives of colonization, white supremacy, and other forms of violent encounter, both for those who bore the brunt of that theft and for those who inherit its rewards.

A body’s relative capacity to be ‘alive’ to movements made on it has historically been bound up in racialized animacy hierarchies, wherein whiteness emerges as the apex of this impressibility (Schuller, 2018: 27). Drawing from Schuller’s theory of sentimental biopower, I argue that the contemporary occult operates as a biopolitical affect regime wherein sensitivity, or the capacity to operationalize impressions from the environment, is a relative rubric that ties populations differently to a desirable future. In other words, learning how to become more sensitive, or receptive, works as the index of civilizational health. How does magic/psychic healing/occult represent an attempt to build capacity for receptivity among participants, and how is this capacity linked to the healing of white supremacy and colonization? In what ways does queerness offer – in the words of one informant – a ‘boot camp’ for building this capacity, and are there different stakes for white people and/or settlers to undergo this process? By framing magic as a process of training which is linked to the affective, I hope to show that contemporary practitioners both *contribute to and disrupt the legacy of sentimental biopower*, maintaining affectability as the locus of social progress at the same time that whiteness is displaced as its coextension. While sensitivity is considered something that can be learned, *queerness*, rather than whiteness, emerges as a predisposition toward this learning, while white supremacy is figured as that which must be overcome.

In this article, I begin to develop a theory of ‘bottoming’ as a prerogative that is particularly salient to white informants, in order to unlearn and thus repair what they see as the fallout of white supremacist legacies, including settler colonialism, capitalism, and environmental degradation. Listening, submission, reception and building the capacity to sit with unpleasant sensations came up in interviews as different strategies of allyship, which magic provides a workshop for. While racialized informants in my study are more likely to discuss their practices in terms of reclaiming what was stolen from them, white participants tend to frame their ‘work’ as a process of building capacity for discomfort. What is consistent across all participants, white or otherwise, is that magic is part of a process of training oneself to unlearn what we have inherited from an unliveable world and learn to attune to something else. Many claimed this attunement was particularly available to them as a result of their experience of social dislocation as queer people.

## Magic as (re-)attunement

Throughout my interviews, one major theme that emerged was the notion that magic helps one attune to normatively hidden patterns. Many described their practices in terms of ‘opening’ or ‘receptivity’, which brings the querent into connection with a pedagogical undercurrent. Some descriptions include ‘Magic . . . is a way to show openness, to receive’; the ‘tuning of your intuition’; ‘alternative sources of power and energy’; and ‘open[ing] yourself up to . . . therapeutic intervention’. In defining the umbrella of practices that might be called occult, one informant claimed,

I think what unites a lot of different practices that fall under this approach is like an attunement to energy. And then each practice is kind of like a different toolset or a different language [. . .] set of practices [. . .] of defining or understanding or working with these energies that are like the unseen, unknowable. (Group interview June 2022)

Participants described the ability to cultivate a receptivity to energy, signs, or teachings as part of the process of learning magic, which has the potential to bring into being a social nexus that has been repressed, or prevented from appearing at all. When recounting how she got into magic, one informant explains, ‘I was taught that this was the world, and it’s actually another world that I relate to, so is there anything else? Is there anything else that has been [. . .] hidden from me?’ (Group interview July 2022). The ‘unseen’, ‘unknowable’, or ‘hidden’ here is a political allegation, wherein any given field of vision is the result of that which has been (incompletely) suppressed or rendered unintelligible by some disciplinary power. Literally, something has been occulted, or concealed. For many informants, their own experiences gave them justification to believe that there is value to that which has been repressed, naming queerness itself as a struggle against secrecy, opacity, and invisibility. ‘I’m much more interested in the reality we don’t have a lot of access to’ (Interview, April 2022).

Gayatri Gopinath (2018) argues that queer aesthetic practice involves a ‘retraining of our vision and re-attunement of our senses, [which] in doing so points to the limits of the entire apparatus of vision that is the inheritance of colonial modernity’ (p. 8). I argue that according to this hermeneutics of misrecognition, wherein something exists ‘out there’ but is not available through the apparatus of vision we have inherited, magic emerges as a form of training to become receptive to the unseen:

These tools, you know [. . .] they invite things that seem inexplicable into these stories we’re trying to create about ourselves. It kind of like, connects you to remembering that even though [. . .] I haven’t had my truth reflected back to me, it’s out there and *I might not be even ready to recognize it*. (Interview April 2022, my emphasis)

Here, the speaker implies that recognition can be learned, granting access to what is currently imperceptible. As I will explore later, while the ‘truth’ that is sought out was not uniform among informants, queer identity figured largely as an example of something which needs new stories to exist within, which involves making what appears natural come into relief as a fiction. ‘One definition, one interpretation of reality has been normalized, and hyper-normalized [. . .] [Magic helps] penetrate another realm, that’s not so awful’ (Group interview, April 2022). Magic conditions one to recognize what already exists, but has been erased or relegated to the margins, where ‘re-attunement’ offers a means to heal from personal and collective injury.

Alex Owen (2007), writing about the popularity of occult and esoteric movements in early-20th century Britain, argues that the intense plurality of animal magnetism, spirit communication, astral projection, and other forms of ‘enchantment’ represented a crisis of method rather than belief, wherein practitioners sought out strategies for adapting to the shifts of a modernizing world. Scholars of Western esotericism and Victorian science have pointed out how, rather than representing a paradox, the persistence of ‘irrational’ occultism in the era where the modern became a horizon of expectation and desire represents a struggle for power between emerging fields of inquiry (Luckhurst, 2002), and the status of illegitimacy justified the subjugation of particular peoples within the shifting boundaries of the American and British empire (Josephson-Storm, 2017; Richardson, 2017). Despite its historical ambiguity, the occult has become crystallized as the constitutive outside of the modern, a relationship that is actively invested in both by debunkers and scientific purists as well as those who practice magic themselves (Hanegraaff, 2014; Luhrmann, 1989). I bring this up here in order to demonstrate how the taken for granted ‘countercultural’ bent of the occult is linked to a theory of history in which the past comes to represent the enchanted detritus of modernity, available for practitioners to reclaim in order to imagine alternative routes of the social. As I have argued elsewhere, even if informants do not see themselves as unearthing an historical practice, the occult is inherently a historiographic concept because of its relationship to the modern, which only becomes legible through relegating certain bodies, knowledge, and lifeways to a spatial and temporal elsewhere (Sheedy, 2022; see also Ogden, 2018; Richardson, 2017). Feminist, postcolonial and other critiques of modernity as a project have conceived of it as ‘haunted’ by what it must repress in order to emerge as a universalizing narrative (Harding, 2008). Postmodern critique is preoccupied with the modern as defined not through inevitable loss but through forced disappearance of certain individuals, things or ideas (Gordon, 2008). In other words, the modern is a process of normalization which cannot be understood outside of empire. While informants had different names for it, many defined the occult in a way that put it in conversation with the modern as a disciplinary technology. Speaking about their own coven, one young white informant said the following:

A lot of us are interested in [astrology] *as a way to like disrupt this idea* that in order to believe in something, it has to be something that can be empirically proven or something that lines up with the traditional scientific rational world view [. . .] People do have a lot of resistance to it and feel this compulsion to convince [me I am wrong]. Why do you feel the need to convince other people to subscribe to your worldview? [. . .] I think it’s kind of like connected to that like . . . I guess more like ‘masculine’ way of thinking [that was] a *huge driving force for imperialism and colonialism*. (Interview, April 2022, my emphasis)

This informant locates astrology within an imperial struggle wherein one ‘worldview’ becomes overrepresented by force. To re-attune to something else connotes a temporal movement, in connecting with something that has been relegated to the past by virtue of its expulsion from the chronology of modernity. In *Unlearning Imperialism*, Ariella Azoulay (2019) has written that ‘retraining our modes of relating to figures in the past involve the critical engagement with what kinds of destructive forces distinguish particular worlds as “past” in the first place’ (p. 21). This temporality, I argue, is key to understanding the historical stakes at the heart of this phenomenon, wherein some prior framework exists as a precedent for informants’ actions, giving weight and authenticity to claims, at the same time that it sets up a tension where making alternative ‘roots’ on settled land carries with it the mark of suspicion. While the term ‘occult’ is often used in reference to formalized esoteric and ritual magic, in this study, it is meant to communicate its original Latin meaning, that something is ‘hidden’. I find this useful given the ways that whatever is ‘hidden’ is conceived of in interviews as always already an effect of power. Apprehending modernity as a vehicle and an effect of violent obfuscation, I argue that my informants find in occultism fertile ground for critiques of boundaries of systematic knowledge, as well as a modality to make perceptible that which has been covered over by its destructive, but never totalizing, force.

## Affective expansion as social transformation

Querents imagine themselves to be part of a social physiology that they participate in through the expansion of their affective capacity. Magic, as re-attunement, not only has the capacity to reconnect us with what was at one point severed, but it is *always already* a critique of what produced that severance in the first place. My informants reflect on this capacity for attunement as a motivator of social cohesion, personal empowerment, or reparation, and often explicitly link their practices to demands for social and political transformation. This could be as modest as ‘constructing moments of intimacy’, to overturning capitalism and reversing climate change. Simply entertaining the idea that our current structures of power are not inevitable was often held up as a foundational political ethic. For example, one informant claimed: ‘What I appreciate about magic is that it’s also a space where we can pray and wish for and speculate and imagine and visualize something else . . . those priorities are decolonization, which is essential to environmental justice’ (Interview, April 2022). One’s sensitivity, or capacity to recognize and tune into impressions that exist in the environment, is heralded as an imperative for political transformation. Attunement, receptivity and openness operate as processes of transcending social barriers in order to access potentialities for living which are otherwise foreclosed.

It is this capacity to receive impressions from the environment: to affect and be affected by the world in ways that bind one to a more liveable, alternative future, which I situate within Kyla Schuller’s theory of sentimental biopower. In *The Biopolitics of Feeling*, Schuller (2018) turns to race science in the 19th-century US in order to theorize an account of biopower that she calls the ‘sentimental politics of life’ (p. 4), wherein populations were (and, she argues, continue to be) organized according to a perceived differential capacity to regulate feeling. She argues that race emerged in this period not as a stable or purely biological inheritance, but a differential capacity to change over time, distinguishing populations according to their relative ability to operationalize impressions received from the environment. Critical of how some affect scholars overstate the liberatory potential of affect, Schuller demonstrates how lively matter actually operated as a key technology of biopower in the 19th-century America, wherein a body’s capacity to incorporate and discipline different kinds of feeling were central to its political claims to life. Sentimentalism, rather than an aesthetic or apolitical mode, worked as a technology to circulate and regulate feeling through a milieu, and reform movements (even ‘radical’ ones like abolition and suffrage) worked to cultivate and maximize this capacity (Schuller, 2018: 18). The populations that were ascribed mutability and could be reformed over time, were differentiated from those who could only pollute, similar to Hortense Spillers’ notion of the ‘mere flesh’ that has historically categorized Blackness in America (Schuller, 2018: 118). As such, affect is always already racialized: the capacity to affect or be affected is itself biopolitical.

If biopower designates certain bodies as indicative of civilizational health, while others are constructed as detrimental to it (Puar, 2007), sentimental biopower demonstrates how apparently benign attributes such as ‘sensitivity’ and ‘receptivity’ actually map out racial governmentality (Nyongó, 2009). Thus, in centering affectability as a key measure of social progress, contemporary occultism emerges as a biopolitical affect regime. The expansion of the affective register, and the capacity to learn how to become sensitive to impressions (construed as energies, patterns, or forces that exist external to oneself) is foundational to informants’ understandings of magic and its political potential. Impressibility itself is heralded as a civilizational marker, wherein social ills can be repaired, if only one learns how to tune into another realm. One informant defined magic as a means to ‘recognize the energy and stuff that are around you. And the fact that you can infuse in them’ (Group interview, July 2022). Another claimed, ‘I have a very connected spiritual worldview where I feel like I’m like in tune with the universe and I can affect and be affected by the world around me and this cosmic beyond material way’ (Interview, April 2022). Finally, ‘[I’m] trying to pay attention to the world feeling like a magical place that’s always giving me information [. . .] depending how receptive I am’ (Group interview, July 2022). In these examples, spiritual enlightenment dovetails with affectability as a conduit and measure of the good. The capacity to affect and be affected by the world: becoming receptive to the information that is ‘out there’ and operationalizing it, is heralded as the foundation of personal actualization and social change. ‘My number one motivation for healing, which I use magic for, is [. . .] to become more resilient, and more *efficace*, efficient, and stronger, in order to participate in movement building’ (Interview, April 2022). Magic is a form of impressibility because it privileges capacity for transformation as a desirable, because reformist, attribute.

Analyzing the place of impressibility in magic, and reading impressibility through the lens of biopower brings into relief the stakes of the contemporary occult, framing race not in terms of a set of cultural possessions, but rather as a conception of differential inherited capacity or range of motion. In the animacy hierarchy that Schuller argues categorizes each race’s relative achievement of civilization, whiteness is most proximate to or aligned with a desirable future. Impressibility serves as the

key measure for racially and sexually differentiating the refined, sensitive, and civilized subject who was embedded in time and capable of progress – as well as in need of protection from the savage elements of the population suspended in the eternal state of flesh. (Schuller, 2018: 8)

Race was both the cause and effect of the body’s variable receptivity, with individual embodiment linked to the collective population over time. Put differently, whiteness operated as both the evidence of heightened capacity, as well as the conduit toward ever-greater health. Porosity, or the body’s receptivity to sensation, operated as a mark of its vitality and futurity.

If queer practitioners of magic are in some ways replicating a biopolitical logic of affect, wherein the circulation of affect operates as an index of civilizational health, do they necessarily maintain whiteness as the marker and conduit for that affectability? As I will elaborate below, while affectability remains legible as a marker of social viability, informants in my fieldwork named queerness, rather than whiteness, as its privileged conduit.

## Queerness as inherent capacity for sensitivity

‘Astrology has always been, well, kinda queer’ (Dockray, 2018). While the resurgence of witchcraft, astrology, and related mystical practices extends much beyond queer people, queer commentators, bloggers, and writers have been eager to establish a particular affinity among them and their kin (Beusman, 2015; Lynch, 2016). Chani Nicholas (2016), queer astrologer with over half a million Instagram followers and author of *You Were Born for this, Astrology for Radical Acceptance*, argues that

queer, trans, and gender nonconforming folx, and many marginalized communities [. . .] have always been attuned to wisdom traditions, art practices, mythologies, and story-telling that explores the value of life beyond the normative conditions we’ve been given, but cannot exist within joyfully.

What makes queer people especially capable of attuning to these wisdom traditions? If the occult becomes, paradoxically, legible through that which was repressed, it necessitates creative methods of encounter in order to access it. I argue that the development of sensitivity functions as this method of encounter, wherein informants work to make themselves open to other worlds. Queerness, construed by informants as a relatively heightened sensitivity, offers access to the occult as a constellation of knowledge encounters that have been marginalized, whose unearthing is key to ushering in a more livable social and political paradigm. Read in the context of sentimental biopower, sensitivity is not an apolitical mode or individual emotional quality, but a highly vexed nexus which has historically organized populations according to different levels of productive mutability. As I hope to show here, participants reify sentimental biopower by maintaining sensitivity as an index of reform, which privileges queer people as more relatively evolved than others. At the same time, this animacy hierarchy is uncoupled (though not completely) from race as its organizing framework. One two-spirit informant from the Anahuac valley (‘Mexico’) described it this way:

People in my community [queer people] have an extraordinary level of sensitivity. [Let’s] call it a gift [. . .] I was more aware about this sensitivity, especially when I didn’t fit with this heteronormativity, patriarchy and so on. Probably the sensitivities you can inherit it. But it is also something that can be learned, right? To develop this sensitivity. And this ancestral philosophy [in Mexico], they say we all have the capability. To heal ourselves and to assist others to healing. (Interview, May 2022)

I want to point out three things in this excerpt: the participant links sensitivity to marginality, refiguring woundedness into a special capacity. Whether sensitivity begets queerness or the other way around, they theorize the correlation between queerness and the occult in terms of affect. Second, the speaker believes that while there is probably some predisposition toward this affective capacity, it is *also something that can be learned*. Third, this heightened sensitivity is maintained as key to healing self and society.

While each informant had different ways of explaining or theorizing what, if anything, makes the occult ‘queer’, most drew a parallel between the structural locations of both objects. Participants described the queer-occult correlation in terms of an ontological position: magic is a marginalized knowledge practice that is not valued, which is akin to queer identity and behavior:

To practice magic is inherently queer because it defies . . . a straight world order that says things are a certain way and there are these certain like boxes that are like the truth [. . .] Further, queerness is inherently magic because it challenges, in capitals Our Reality which would . . . like *if we didn’t have restrictive systems, we wouldn’t have like the word queer, right?* (Interview, April 2022, my emphasis)

In this example, the speaker recognizes that queerness cannot be understood outside of a relationship to power: it is not simply a descriptor of sexual or gendered behavior, but rather functions as a form of ‘defiance’ of ‘reality’ or a ‘straight world order’. This is where its kinship with magic lies, as a putative shared opposition or potential opposition to what is hegemonic.

Not everyone saw queer and magic as an innate pairing: for example, some pointed out that ‘it’s not unique to being queer’ to have an experience of marginalization that might push you to ‘seek answers that go deeper’ (Interview, May 2022). Others argued that the occult and queerness are ‘not *necessarily* opposed but *currently* opposed [to] . . . the dominant hegemonic paradigm’ (Group interview, June 2022), and asserted that practices like astrology were not always considered illegitimate. The putative illegitimacy of the occult is key to its reclamation as a queer modality. Many pointed toward the fact that the occult was seen as ‘trashy’, ‘cheap’, or ‘low’ as a kin hermeneutic with queer experience, in that it represents social dislocation or devaluation, which then becomes politically useful as a way to critique the value system which renders it abject.

Participants see themselves as resisting domination by remaining open to that which is normally shunned:

The queer community, we’ve been used to being outsiders, like used to being at the margins of society and maybe it makes us more open to what’s different, and it makes us more fluid and understanding and open to these kind of different things. (Interview, June 2022)

Consistent with Schuller’s biopolitics of feeling, queer ‘fluidity’ is here a privileged attribute, wherein plasticity is offered as a conduit for social enlightenment. Some described it as a skill set that is honed, wherein the experience as a queer person acts as a kind of prerequisite, or a way in, to magic. For one non-binary Afro-Latinx informant,

Being queer is something that exists between so many different realms that we have to navigate on a constant basis. And that experience itself, I feel kind of *prepares someone emotionally and spiritually* to be able to make those sort of connections that are far grander than other people might be able to understand. I feel like there is a spiritual element to being queer itself that, you know, allows people to tap into on experiences and vibes of whatever lie beyond the norm. (Interview, May 2022, my emphasis)

Queer experience is offered in this instance as a form of skill building. Simply moving through the world as a marginalized person apparently facilitates a capacity for attunement, wherein one might ‘tap into’ experiences that are unavailable to others. This notion of special knowledge linked to one’s position within power is pervasive in leftist, critical politics. ‘The very state of being oppressed is somehow supposed to confer a greater clarity of vision, a more authentic view of the world, than the bourgeois trappings of economic, racial, and sexual hegemony’ (Gross and Levitt, 1998: 33). As one trans informant argues,

There’s like this intensity I feel . . . of things fitting and not fitting. And you’re like, since birth kind of forced to think critically about things in your own experience in relation to the world [. . .] I think there’s a critical thinking, [an] intensity and consciousness that comes from being queer. (Interview, May 2022)

Intensity and consciousness is linked to being skilled at magic, and queerness operates in these instances as a process of training, where one develops a capacity for openness. Put differently,

[Being queer] is like a boot camp! And not to say that inherently people who are queer are going to be more open to like spirituality or any of these things. But I feel like if somebody kind of leans into that experience [. . .] that journey will lead them to being able to make these larger connections, spiritually. (Interview, May 2022)

Informants perceive themselves as more open, receptive, or attuned to the elsewhere that we named in interviews as the occult, with this receptivity figured as a positive good. Wherever the sensitivity comes from, whether an ‘intense consciousness’ that queer people are born to use, or something that one has fostered to by virtue of navigating a hostile world, most informants saw it as something that can (and should) be cultivated, or learned.

## Bottoming as an antidote to white supremacy

If sensitivity can be learned, and this sensitivity is conceived of as the heart of social transformation, how does magic offer a kind of workshop for that learning? The goals or motivations for building a spiritual practice varied between participants, including healing childhood trauma, navigating homo- and transphobia, connecting to nature, and surviving sexual assault. However, the most common theme that emerged was the healing of intergenerational trauma. ‘Why I practice magic is because I have so much to heal, so much trauma from generations and I need to understand it and heal it I need to flip it’ (Group interview, July 2022).

The focus on genealogies of violence, or inheritance, and the ways in which this is tied to race in my interviews came out of what exploratory fieldwork had already shown to be a major motivation for many people involved in this ‘movement’. The rise of cultural witchcraft, mystical and occult media has been accompanied by advice on ancestral healing for white people (Peacock and Germaine-Strickland, 2018), acknowledging the ‘witch wound’ as a form of collective harm (DaSilva, n.d.), the rise of Black and Latinx witches channeling their ancestral magic (Long, 2021) and books on spiritual activism (Ricketts, 2021), including titles like ‘White Magic’ (Washuta, 2021) and ‘Postcolonial Astrology’ (Kat, 2021). The focus on intergenerational trauma looms large in the cultural imaginary in general, as people increasingly come to terms with how we are entangled with the ongoing legacies of slavery, dispossession and genocide. In settler colonies like Canada, intergenerational trauma is readily used to unpack the legacy of the residential school system and other architectures of (always resisted) Indigenous assimilation and removal. If the figure of the witch has re-emerged as part of a negotiation with folk knowledge and their relationship to power, many also offer a critique of how witchcraft and other psychic healing work has also been used as a fantasy of transcending race (Morgensen, 2011), and are anxious about how not to reify this fantasy. New age spirituality came up consistently in interviews as an apparent foil to the work that participants are undertaking, criticized as being hollow, capitalistic and appropriative. Participants were often very quick to differentiate their own practices from ‘Texan white ladies with their sage’, and professed the wish to, in the words of one white informant, ‘participate in resistance from my own position’ (Interview, April 2022), as opposed to veering into cultural theft.

As an allegory for the modern, the occult is a deeply temporal category which becomes legible through the relegation of certain life ways, knowledges and populations to the past as illogical ‘survivals’ of modernity and its domestication of otherness (Josephson-Storm, 2017). The occult is foremost a knowledge relation: ‘the dark canvas of presumed otherness modernity needs in order to paint the outlines of its own identity’ (Hanegraaff, 2014: 254), wherein certain modalities of knowing and being in the world are disciplined against the apparently ‘mythless’ educated, urban, elites of the Occident (Richardson, 2017). Returning to Azoulay, the occult can be read as that which has been banished to the ‘past’, a forced disappearance that is mythologized as inevitable. This temporal structure means that magic is always already a kind of revival (Josephson-Storm, 2017). As such, notions of inheritance, genealogy and legacy are foundational to contemporary occult formations, and key to understanding how race emerges within them.

If whiteness is an ideology inextricably linked to empire and the obsoletion of that which is racialized against it, how can it be disavowed? What would become available as a lineage, and how could this be claimed without co-opting something one is not entitled to? While my fieldwork is in no way limited to the experiences of white people, whiteness emerges as particularly salient in my project in terms of how whiteness has differently recruited populations into its machinery, and to what extent that ongoing process is resisted and opted out of. While almost all informants sought out a means to heal from a perceived rupture or severance, I focus here on the ways that redress emerged as fraught for white people or settlers, who also felt guilt as the inheritor (even perpetrator) of that violence. If the occult represents a temporal, and thus racialized, modality, and whiteness garners its power through a putative cultural neutrality (Lipsitz, 2018), almost all magic or occultism appears as necessarily culturally transgressive for those who are white. As such, the risk of the slippage into cultural appropriation is a constant negotiation in psychic healing, *even for, and sometimes* ***especially*** *for those who are critical of their inherited power* through white supremacy. Given the ways that white witchcraft or magic remains, with good reason, highly suspect in critical explorations of this phenomenon, it is necessary to explore how that risk is navigated by white informants themselves.

So far we have seen how the occult, as a repressed or invisible modality, requires the cultivation of sensitivity in order to apprehend it, and how informants understand queerness as a predisposition to developing this capacity. In this final section, I want to theorize the capacity to ‘bottom’, or become radically receptive, as the prerogative of white people as an antidote to histories of infringement and violent confrontation that informants see themselves as descendants of, and wish to overturn. The term *bottoming* as a way to conceive of the reversal of white supremacy comes from one of my white, trans interviewees:

Bottoming is like this courage to open and receive. And submit. The actual willingnesss to do all those things [. . .] like actually step into bottoming is absolutely connected with other forms of emotional intelligence and wisdom [. . .] White supremacy needs to surrender and submit, in this bottoming and this service way, and needs to find pleasure in submitting, in surrendering. (Interview, April 2022)

I find the metaphor of bottoming apt to imagine the ways that receptivity, queerness and white supremacy interact in this study. If the occult is construed as a form of sentimental biopower which privileges receptivity as a force for social change, white informants articulate this receptivity in terms of listening to Black, Indigenous, and people of color (BIPOC) teachers, sitting with difficult sensations, inviting discomfort and opening oneself up to accountability. While receptivity is maintained as integral to civility, informants make a reversal of the animacy hierarchy Schuller outlines in 19th-century race concepts. Whiteness, rather than operating as the apex of this receptivity, is figured as that which is in need of reform.

In our group discussions especially, participants talked about strategies or tensions of anti-racist work and the place of whiteness in witchcraft or magic, theorizing how to be in relationship with knowledge traditions that might be pedagogical. Humility and awareness came up often, with listening and learning prioritized as forms of repair:

If you’re trying to approach things from different cultures, I think just trying to do it as holistically as possible and know your relationship to that thing [. . .] that maybe your role will always be in a role of submission and learning and like receptive. (Group interview, June 2022)

Here we can see how magical traditions are referred to as ‘different cultures’, against whiteness as that which has been voided of such objects. One white informant spoke about whiteness as a ‘disorientation’:

[W]e haven’t exactly figured out how to be involved in comfortable ways. So a lot of the time to be in dialog with a culture that’s not yours – especially if you’re a white person and you’re talking about a marginalized culture – it’s to stay in your own discomfort at the fact that this culture was maybe erased because of your ancestors, and *that learning that is actually a sacred practice to like help undo things* [in] previously violent situations. [. . .] There are ways to be in relationship with that *if you’re willing to be humble and receptive.* (Group interview June 2022, my emphasis)

Here, learning to become receptive is a way to undo violence, to reach the ‘threshold of caring’ that they argue white supremacy obscures. Bottoming has been explored in queer theory as a pleasurable surrender of power, even an ethical mode of relationality (Hoang, 2014). Thinking bottoming in terms of receptivity – as opposed to passivity – is a critical modality in that it represents a submission of authority to make possible different forms of connection, even solidarity, with others.

Another white informant described this goal slightly differently: ‘There’s a necessity to facilitate people being inside of their bodies. And to trust their bodies and to build capacity for unpleasant sensations’ (Interview, May 2022). By making oneself radically open to different ways of being in the world, one has the potential to access different ways of mapping the social, and magic offers a conduit to this openness. The transformative energy, or teaching, is sometimes construed as coming from the ‘universe’ at large, or from other divine mediators. ‘Magic is a way to show openness . . . to receive their teachings’ (Interview, April 2022). For another white, first generation Canadian interviewee, this guidance comes from other people, and offers an inroad to fostering solidarity with them:

[Tarot] is a way of training the way that I think, to see differently as well. And I think *it comes from a desire to see otherwise [. . .] to see beyond colonial institutions*. I think experimenting with different forms of thinking and forms of otherness and forms of acting and like disturbing the status quo [. . .] *creates spaces in myself* for different types of disruptions. [. . .] Making your sense of reality fluid, I think can only serve to allowing yourself to not impose belief systems *[. . .]* upon others as well. I try to be very, like, *receptive to a situation and to, what I learn from people of color and Indigenous people* and that, like humbleness of always learning I think is also something that I’ve developed through my spiritual practice. (Interview, May 2022, my emphasis)

The speaker designates their spiritual practice as a form of training: one’s ‘sense of reality’ is subject to change by learning from people of color, enacting a personal and eventually a collective social transformation. This transformation requires, and itself comprises, divestment from colonial systems: a direct example of Gopinath’s (2018) disinheritance of colonial modernity’s field of vision. They use terms like ‘fluidity’ and ‘receptive’, as well as describe the process as ‘creating spaces for disruptions’, which privilege plasticity, receptivity and sensitivity as key qualities of personal and social reform.

On bottoming, Bersani (1987: 217) has argued that at the same time that one may identify with masculinity, there is immense pleasure in violating its authority, instead embracing a disintegration of the self. Thinking of this abdication of power in terms of whiteness provides compelling grounds for theorizing divestment and reparative solidarity through the lens of a queer relationality. Bottomhood makes possible a ‘radical Elsewhere’ (Hoang, 2014: 2) which my informants consider urgently necessary, and condition themselves to access by disidentifying with the forms of power they argue are obstructing it.

## Conclusion

Receptivity is not a benign category, but has historically been associated with the differential organization and ranking of populations based on a perceived capacity to evolve (Schuller, 2018). While informants in this study framed white supremacy as undesirable, and worked to oppose it, the maintenance of animacy as an index of social progress presents a conundrum that gives some new insight into the potential slippages of the occult that critics point out as ‘suspicious’ (Cornum, 2018). Indeed, the radical ‘openness’ that informants laud as a conduit toward divesting from whiteness is ironically already implicit in psychic healing movements, wherein white women especially have been framed as porous conduits for spirit channeling, necromancy, astral projection, or otherwise connecting with some higher power which the human race requires for a paradigm shift (Cox, 2003; Crowley, 2011). Remaining open and especially sensitive to external forces has also meant transcending barriers of race in order to ‘call in’ Indigenous spirit guides and achieve a colourblind harmony that absolves the seeker of their earthly shackles and finds more genuine roots on stolen land (McGarry, 2012; Troy, 2017). While informants privilege queerness, and not whiteness, as offering a particular sensitivity for this work, therefore making a revision of the sentimental biopolitical framework, the availability of the language of animacy within the legacy of spiritual healing is so embedded within racialized concepts of collective health that it demands more explanation of what kind of satisfaction practitioners might be getting from its usage.

In this article, I have argued that the queer occult represents a significant contemporary phenomenon, whereby people looking for alternative maps for living find not only an imagined precursor for resistance to our current normal in magic, witchcraft or psychic healing, but also the means to develop forms of attunement to a potential otherwise. By investigating the occult as a method of making kin with that which has been repressed, I theorize how queerness may offer a particular kind of allowance for claiming that kinship. By drawing connections to sentimental biopower, I locate the queer occult within biopolitics, wherein the expansion of sensitivity offers a conduit for social transformation. Making this interdisciplinary maneuver brings into relief the racial stakes of the occult wherein opening oneself up to knowledge traditions that have been severed or incorporated into whiteness is framed as a requirement for betraying white supremacy, at the same time that it risks maintaining white interiority as the outcome of the civilizing drive (Castiglia, 2002).

What is necessary, in the field of spiritual healing or esotericism, as in any work that explores allyship, decolonization, and anti-racism, is to critically engage with the ways that betraying white supremacy involves divesting from some lineages in favor of others. If contemporary occult movements represent a turning toward putative repressed or devalued modalities that may rival those which we have otherwise inherited, queer informants theorize their special relationship to these objects through a framework of sensitivity that magic allows them to workshop. ‘Bottoming’ is offered as a strategy for surrendering power, which has the potential to overturn centuries of imposition and trespass, which white people in particular recognize as work that they need to do.
